# Occupational Exposure to Volatile Organic Compounds (VOCs), Including Aldehydes for Swedish Hairdressers

**DOI:** 10.1093/annweh/wxac078

**Published:** 2022-11-24

**Authors:** Niklas Ricklund, Ing-Liss Bryngelsson, Jessika Hagberg

**Affiliations:** Department of Occupational and Environmental Health, Faculty of Business, Science and Engineering, Örebro University, SE 70182 Örebro, Sweden; Department of Occupational and Environmental Health, Faculty of Business, Science and Engineering, Örebro University, SE 70182 Örebro, Sweden; Department of Occupational and Environmental Health, Faculty of Business, Science and Engineering, Örebro University, SE 70182 Örebro, Sweden

**Keywords:** chemical mixture, hair treatment, hazard index, limit values, maximum cumulative ratio, reference values

## Abstract

Working as a professional hairdresser involves the daily usage of many different hair treatment products containing chemicals in complex mixtures. Exposure may induce symptoms in the airways and on the skin. In this study, exposure of hairdressers to volatile organic compounds (VOCs), including aldehydes, was measured in the personal breathing zone in the spring of 2017. The study included 30 hairdressers evenly distributed over ten hair salons in the town of Örebro, Sweden. Work tasks and indoor climate were also surveilled. A hazard index (HI) based on chronic reference values for health was calculated to indicate combined exposure risk. In total, 90 VOCs, including nine aldehydes, were identified. Individual exposure expressed as a total concentration of VOCs (TVOCs) were in the range of 50–3600 µg/m^3^ toluene equivalent (median 460 µg/m^3^) and the HI was in the range 0.0046–13 (median 0.9). Exposure was more strongly influenced by variability among hairdressers than among salons. The HI indicated an increased risk of non-carcinogenic effects (HI ≥ 1) at four of the 10 hair salons. Individual working procedures, ventilation, volumetric usage of hair treatment products, certain chemicals in products (formaldehyde, isopropanol, and 2,4- and 2,6-toluene diisocyanate), and availability of reference values may have affected estimates of exposure risks. Nevertheless, the HI may be suitable as a screening tool to assess potential exposure risk posed to hairdressers since it considers the complexity of chemical mixtures and the chronic component of VOC exposure occurring in all indoor environments.

**What’s Important About This Paper?**

Hairdressers use a variety of products during their work that result in exposures to complex chemical mixtures. This study characterized personal exposure to more than 90 volatile organic substances (VOC) including aldehydes, and found that exposures varied and were strongly influenced by daily work activities. The hazard index for some hairdressers was dominated by single substances. This study contributes to the evidence base that hairdressers are in need of strategies to reduce chemical exposure risks and improve working conditions in hair salons.

## Background

Approximately 24 000 people work as hairdressers in Sweden, a common profession. Hairdressing involves the daily usage of many different hair treatment products. These products are composed of a variety of chemicals in order to obtain different performance characteristics, e.g. color, viscosity, moisturizing, and film-forming properties. Usage of chemicals in hair treatment products on the European market are restricted by the Regulation (EC) on Cosmetic Products No 1223/2009 (European Commission, 2009).

Occupational exposure of hairdressers to chemicals from hair treatment products may occur via the skin and airways. Skin on hands and arms is exposed to wet work, as well as to irritant and sensitizing chemicals from activities like dyeing and bleaching. Hand eczema from hairdressing is common (Havmose *et al.*, 2022). The exposure via skin can to a large extent be avoided by correct usage of protective gloves (Havmose *et al.*, 2020). Exposure via airways to chemicals from hair treatment products may occur from the hairdresser’s own work tasks, indirectly through colleagues’ work tasks, or by emissions from the storage or disposal of products in the salon. Therefore, the indoor air of the hair salon is a complex mixture of chemicals. This working environment implies an increased risk for health effects in the airways, but reproductive effects and endocrine effects may also be of concern (Quiros-Alcala *et al.*, 2019). Furthermore, associations between hairdressing and cancer have been shown. In a comprehensive meta-analysis, it was concluded that hairdressers have an excessive risk for multiple myeloma and cancer in the lung, larynx, and bladder (Takkouche *et al.*, 2009).

Exposure of hairdressers via indoor air in hair salons has been examined in several previous studies with a focus on volatile organic compounds (VOCs) (Ronda *et al.*, 2009; de Gennaro*et al.*, 2014). VOCs are a diverse group of chemicals that includes solvents, preservatives, fragrances, and propellants emitted from most hair treatment products. Furthermore, studies of hairdressers have also considered exposures to persulfates from bleaching (Mounier-Geyssant*et al.*, 2006; Nilsson *et al.*, 2016); ammonia related to permanent hair dyes and wave preparations (Mendes *et al.*, 2011; Nemer *et al.*, 2015); formaldehyde from hair straitening products (Chang *et al.*, 2018; Asare-Donkor*et al.*, 2020); aromatic amines like phenylenediamine and diaminotoluene from permanent hair dyes (Hueber-Becker*et al.*, 2007; Helaskoski *et al.*, 2014); hydrogen peroxide from lightening and permanent hair dyes (Mounier-Geyssant*et al.*, 2006); thioglycolates from wave preparations (Oikawa *et al.*, 2012); and phthalates used as fragrances or solvents (Kolena *et al.*, 2017; Chang *et al.*, 2018).

Mixtures of chemicals in the indoor air of hair salons, in combination with the broad-spectrum health effects observed among hairdressers, calls for risk assessments adapted to the complexity of the exposures involved. For evaluation of the indoor air quality in hair salons, application of a total hazard ratio indicator was proposed by de Gennaro*et al.* (2014). The total hazard ratio indicator, hereafter referred to as the hazard index (HI), is the sum of quotients of measured indoor air concentrations of VOCs and their corresponding reference values. A reference value (RV), in this context, refers to a concentration below which chronic exposure to a single VOC is unlikely to cause non-cancer health effects, e.g. respiratory symptoms. De Gennaro *et al.* (2014) found an increased potential risk for non-cancer health effects in 11 out of 12 hair salons. Estimates for corresponding cancer risk indicators were mainly dependent on chemical contributions from traffic emissions outside, which is why the HI was less suitable for this purpose (de Gennaro*et al.*, 2014). The HI approach is in line with recommendations in the World Health Organization/International Programme on Chemical Safety (WHO/IPCS) framework concerning a general methodology for risk assessment of combined exposure to multiple chemicals (Meek *et al.*, 2011).

The objectives of the present study are to measure the personal exposure of hairdressers to VOCs, including aldehydes, at Swedish hair salons, estimate non-cancer exposure risk through the application of the HI, and clarify the relative contribution to exposure from single substances.

## Methods

In the spring of 2017, recruitment took place for 30 hairdressers evenly distributed over ten hair salons in the central town of Örebro, Sweden. Exposure measurements were performed during one work shift and comprised of VOCs, including aldehydes, in the personal breathing zone of the hairdressers. Measurements of physical parameters affecting the indoor air quality in the hair salons were also conducted (i.e. ventilation, humidity, and temperature) and work tasks were noted. TVOCs and HIs were derived from chemical exposure data and reviewed RVs based on non-cancer exposure risks or occupational exposure limits (OEL) if no RVs were available. The calculated exposure was evaluated regarding the aggregated and relative contribution of individual VOCs. Ethical approval for the study was granted by the Swedish Ethical Review Authority (decision no 2017/414).

Personal air sampling in the breathing zone of the hairdressers at the hair salons took place during a self-reported easy-to-normal workload and was on average sustained for 176 minutes (CV 6%) of one work shift. Three to five hairdressers were working simultaneously at each hair salon. Work tasks during sampling are presented in Table 1. The hairdressers wore gloves (of unspecified type) during wet work, but no other personal protective equipment was utilized. Sampling was performed on adsorbents (Tenax TA for VOCs and Sep-Pak XpoSure Plus for aldehydes) connected to portable mini-pumps (AirChekXR 5000, Scantec Nordic) carried by the hairdressers. Airflow rates for VOCs and aldehydes were set to 0.05 and 0.5 milliliters/minute, respectively, and repeatedly controlled with a mass flow meter (4140, TSI Incorporated).

**Table 1.** Hair treatments performed by the hairdressers during chemical exposure measurements at hair salons in Örebro, Sweden (spring 2017). Cutting also includes styling.

	Treatments		
Hair salon #	Hairdresser 1	Hairdresser 2	Hairdresser 3
1	Cutting × 3, dyeing	Cutting × 2, dyeing, bleaching	Cutting × 3, dyeing × 2
2	Dyeing × 2, bleaching	Dyeing × 2	Cutting, dyeing, and cleaning the hair salon
3	Dyeing × 2, bleaching	Cutting × 3, dyeing	Cutting × 3, bleaching
4	Cutting, bleaching × 3, dyeing	Cutting × 3, dyeing × 2, bleaching	Cutting × 3, dyeing
5	Cutting × 4	Cutting, bleaching	Cutting × 2, dyeing
6	Dyeing × 2, washing	Washing × 2, dyeing, bleaching	Cutting × 2, dyeing, bleaching, washing
7	Dyeing, washing, and cleaning the hair salon	Cutting, dyeing, washing × 2	Bleaching, dyeing
8	Cutting, dyeing, wig gluing	Cutting, bleaching	Cutting × 2, wig gluing
9	Dyeing, washing × 3	Dyeing, bleaching, styling × 2	Dyeing, washing, styling
10	Cutting, dyeing × 2, washing	Cutting × 2, dyeing	Dyeing × 3, bleaching × 2, styling

The analytical procedures are described in detail elsewhere (Persson *et al.*, 2019) and concord with ISO 16017-2:2003 and SS-ISO 16000-4:2011(International Organization for Standardization, 2003 and 2011). The Department of Occupational and Environmental Medicine laboratory at Örebro University Hospital, where the analysis was performed, is accredited by Swedac, the national accreditation body for Sweden, following ISO/IEC 17025:2017 (International Organization for Standardization, 2017).

Briefly, an analysis of VOCs was performed using an automated thermal desorption system coupled with a gas chromatograph connected to a mass spectrometer. The compounds were ionized via electron ionization, separated on the column, detected in full scan mode, identified from the NIST-2011 MS Library, and quantified using an internal standard of trimethyl pyridine calibrated against toluene, yielding toluene equivalent concentrations with the unit μg/m^3^, hereafter referred to as μg/m^3^ TVOC. Sample blanks were analyzed to determine background levels. The aldehydes, forming aldehyde-hydrazone-complexes with the 2,4-dinitrofenylhydrazin-impregnated silica surface of the adsorbent, were extracted in acetonitrile, separated via high-performance liquid chromatography coupled with an ultraviolet detector, and quantified using external calibration.

Reporting limits of 3 µg/m^3^ and 0.3 µg/m^3^ were used for VOCs and aldehydes, respectively. For VOCs, the reporting limits corresponded to a peak height 20 or more times higher than the background noise in the chromatogram of individual samples. For aldehydes, the reporting limits were equal to or greater than the AM concentration of individual compounds in ten method blanks plus ten SDs of the AM. Concentrations below the reporting limit were not included in the TVOC or the HI.

The indoor climate at each of the hair ten salons was measured in parallel with exposure measurements at a stationary location in the middle of the room, over approximately three hours. For this purpose, indoor climate surveillance equipment from Nordtec Instrument AB, model Testo 480, was used with the logging function of air temperature, the concentration of CO_2_, and relative humidity (RH) every fifth second. The type of ventilation and its location was noted.

### Exposure measures

The TVOC was specified as the concentration corresponding to the summarized peak area of all compounds eluting between hexane and hexadecane in the gas chromatographic mass spectrums acquired from chemical analysis.

The *hazard quotient* (HQ_i_) was calculated by dividing the concentration in the air ($C_{i}$) of each identified chemical ifrom the exposure measurement by its corresponding RV ($RV_{i}$); see Equation 1. The $HQ_{i}$were summed to acquire the HI; see Equation 2. Values of HI>1delimited estimated risk from no risk (HI<1) for non-cancer health effects among hairdressers.


$$\begin{array}{l} HQ_{i}=C_{i}/RV_{i} \end{array}$$ 1

$$\begin{array}{l} HI=\underset{i}{\sum }\;HQ_{i} \end{array}$$ 2

*The maximum cumulative ratio* (*MCR*) was evaluated according to an application for indoor air quality previously described by De Brouwere*et al.* (2014). Calculation of MCR was done by division of *HI* by its highest contributing $HQ_{i}$($maxHQ_{i}$), as in Equation 3:


$$\begin{array}{l} MCR=HI/maxHQ_{i} \end{array}$$ 3

Obtained MCR values were sorted into their corresponding substance groups, defined in Table 2.

**Table 2.** The substance groups of the MCR.

Group	Definition	Comment
I	$maxHQ_{i}>1\;\;\;(HI>MCR)$	*Single substance concern*
II	HI<1	*Low concern*
IIIA	MCR〈2, HI〉1	*Concern for combined effect dominated by one substance*
IIIB	MCR>2, maxHQ<1	*Concern for combined effect by several substances*

The RVs were obtained from De Brouwere*et al.* (2014), in which a structured review process for the selection of data was applied. Updates were checked for in the original databases. For chemicals not included in the compilation, RVs were derived firsthand from recommended databases in De Brouwere*et al.* (2014): governmental databases with established systems for quality control (peer-review processes); transparency; and up-to-date evaluations (conducted within the last five years). Swedish, Nordic, and international OEL were used second-, third-, and fourth-hand, retrieved from the GESTIS database (IFA, 2022). When several optional limit values were available within the categories of Nordic or international OEL, the lowest limit value was chosen. A chemical was excluded from the HI calculations if no reference or occupational exposure limit could be found.

### Statistical analysis

Standard parameters such as the arithmetic mean (AM), standard deviation (SD), geometric mean (GM), geometric standard deviation (GSD), and range were calculated for the exposure measurements. Data were shown to be not normally distributed by the Shapiro-Wilk test. Separate statistical analyses on logged and unlogged data were conducted, showing no differences between the two approaches. Therefore, values on their normal scale were used. Differences in exposure expressed as TVOC and HI between the different salons were analyzed using one-way ANOVA with the Bonferroni post-test. The relationships between TVOC and HI, and concentrations of CO_2_ in the hair salons and exposure, were assessed using linear regression. IBM SPSS Statistics 25.0 was used to perform the statistical analysis. P-values of less than 0.05 were considered statistically significant.

Other data on indoor climate, ventilation, and hairdressers’ work tasks were only qualitatively assessed in relation to exposure levels.

## Results

### Exposure measurements—TVOC concentrations and HI

From the exposure measurements, 90 VOCs, including nine aldehydes, were identified (supplementary material, Supplementary Table S1). A selection of these chemicals, with significant contributions to exposure, is presented in Table 3. The most frequently occurring chemical was acetone, identified in 29 out of the 30 samples. Another 13 chemicals commonly occurred in ≥10 samples, namely: decamethylcyclopentasiloxane (*n* = 27); propylene glycol (*n* = 26); dodecanol (*n* = 22); limonene (*n* = 22); 2-phenoxyethanol (*n* = 21); a vertenex compound (specific structure not available, *n* = 21); formaldehyde (*n* = 20); nonanal (*n* = 18); hexadecanol (*n* = 17); acetaldehyde (*n* = 16); decanal (n=13); dihydromyrcenol (*n* = 12); and hedione (*n* = 10). The rest of the identified chemicals occasionally occurred in <10 samples.

**Table 3.** Selection of VOCs including aldehydes from measurements of exposure in the personal breathing zone of 30 hairdressers in Örebro, Sweden (spring 2017). Concentrations are given in µg/m^3^ toluene equivalents. Criteria of selection: *high detection frequency* (in ≥10/30 samples) and/or *available chronic reference value for health* (RV) and/or *high maximum hazard quotient* (maximum HQ ≥ 0.1), derived from the RV, or if not available, the occupational 8-hr exposure limit (OEL). Presented RVs and OELs were extracted by the procedure given in the Methods section.

Chemical	CAS No.	No. of samples detected (*n* = 30)	Arithmetic mean	Median	Minimum	Maximum	Geometric mean	Geometric standard deviationSD	Standard deviation	Maximum HQ	RV	OEL	Data source
TVOC		30	520	460	50	3600	350	2.8	640				
1-Methoxy-2-propanol	107-98-2	2		13	8	18	12	1.8	7.1	0.001	7000		OEHHA, 2008
2-Buthoxyethanol	111-76-2	3	6.7	6	4	10	6.2	1.6	3.1	0.006	1600		IRIS, 2010
2-Butoxyethylacetate	112-07-2	1	7.0	7	7	7	7			0.05	150		AFFSET, 2009
2-Ethylhexanol	104-76-7	9	30	3.9	2.6	220	6.9	4.4	73	0.4	540		AgBB, 2012
2-Phenoxyethanol	122-99-6	21	11	10	4	19	9.1	1.8	5.4	0.003		5700	IFA (Germany)
2,4-Toluene diisocyanate	584-84-9	1	10	10	10	10	10			0.7		14	IFA (Sweden)
2,6-Toluene diisocyanate	91-08-7	1	12	12	12	12	12			0.9		14	IFA (Sweden)
Acetaldehyde	75-07-0	16	14	7.6	4.7	30	11	2.0	9.5	0.2	140		OEHHA, 2008
Acetone	67-64-1	29	53	37	11	170	42	2.0	41	0.002	70000		Health Canada, 2017
a-pinene	80-56-8	4	10	12	<3	14	8.6	2.5	5	0.03	450		JRC, 2005
Decanal	112-31-2	13	9.6	8.9	5.3	22	8.7	1.6	5.1				
Decane	124-18-5	2	7.5	7.5	5	10	7.1	1.6	3.5	0.002	6000		AgBB, 2012, AFSSET, 2009
Dihydromyrcenol	18479-58-8	12	8,3	7	<3	20	6.8	2.0	5.4				
Dodecanol	112-53-8	22	14	7.5	<3	87	8.7	2.6	19	0.009		1000	IFA (Latvia)
Formaldehyde	59-00-0	20	13	8.8	5.3	38	11	1.9	10	4	9		OEHHA, 2008
Hedione	24851-98-7, 2630-39-9	10	11	5.5	<3	57	6.9	2.4	16				
Hexadecanol	36653-82-4	17	35	8	3	180	12	3.7	60				
Isopropanol	67-63-0	1	58	58	58	58	58			8	7		OEHHA, 2008
Limonene	5989-27-5	22	45	9.5	3	310	18	4.1	70	0.7	450		JRC, 2005
MEK; 2-Butanone	78-93-3	2	6	6	6	6	6	0	0	0.001	5000		IRIS, 2003
Nonane	111-84-2	1	<3	<3	<3	<3	<3				200		IRIS, 2009
Nonanal	124-19-6	18	11	8.2	4.7	28	9.4	1.7	6.8				
Octanal	124-13-0	5	14	12	7.4	20	13	1.5	5.8	0.03	650		AFSSET, 2009
Propylene glycol	57-55-6	26	17	15	3	65	13	2.1	13				
Siloxanes; silicones	14857-34-2	29	197	200	5	700	100	4.3	180	0.3		2100	IFA (Denmark)^a^
Toluene	108-88-3	6	22	22	14	31	21	1.3	6	0.1	260		WHO
Undecane	1120-21-4	2	8	8	6	10	7.8	1.4	2.8	0.002	6000		AgBB, 2012, AFSSET, 2009
Xylene	95-47-6, 108-38-3, 106-42-3	1	<3	<3	<3	<3	<3				200		ATSDR

Acronyms: AgBB = Ausschuss zur gesundheitlichen Bewertung von Bauprodukten, Umweltbundesamt; AFSSET = L’Agence française de sécurité sanitaire de l’environnement et du travail; Health Canada = Government of Canada; IFA = Institute for Occupational Safety and Health of the German Social Accident Insurance, in parenthesis: country/work environment authority related to the designated OEL; IRIS = Integrated Risk Information System, US EPA; JRC = Joint Research Centre, European Commission; OEHHA = California Office of Environmental Health Hazard Assessment.

^a^Dimethylethoxysiloxane.

**Figure 1.**
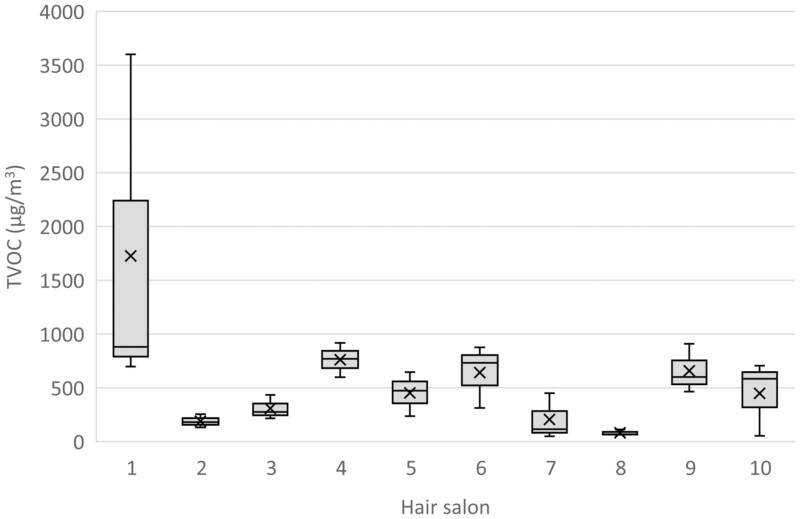
Exposure to TVOC including aldehydes in the personal breathing zone of three hairdressers at each of 10 hair salons in Örebro, Sweden (spring 2017). Concentrations are given in μg/m^3^. Boxplot illustrating minimum–maximum (vertical line); upper and lower quartile (box); median (horizontal line in box); and arithmetic mean (cross).

**Figure 2.**
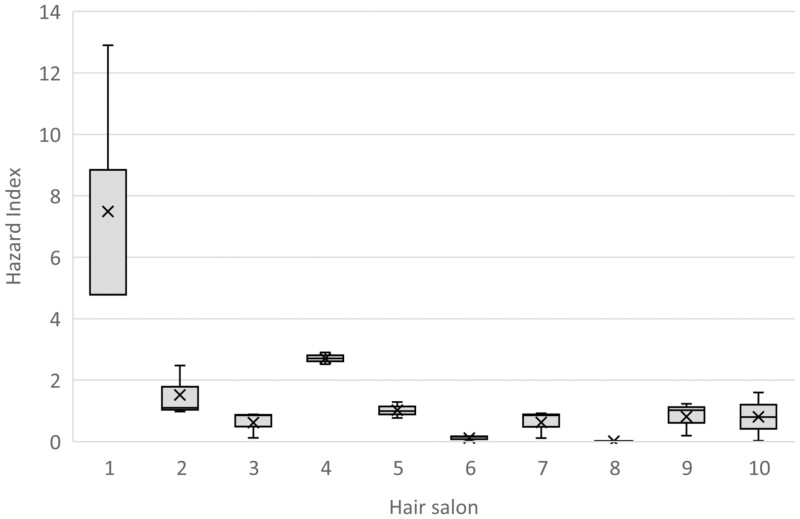
Hazard index of TVOC including aldehydes for three hairdressers at each of 10 hair salons in Örebro, Sweden (spring 2017). Boxplot illustrating minimum–maximum (vertical line); upper and lower quartile (box); median (horizontal line in box); and arithmetic mean (cross).

For all hairdressers, measured exposure expressed as the TVOC concentration was in the range of 50–3600 µg/m^3^, with median of 460 µg/m^3^ (Fig. 1). The AM exposure of the TVOC in each hair salon was 550 µg/m^3^, with a range of 81–1700 µg/m^3^. Differences in the mean TVOC concentrations between hair salons were not significant (*P* = 0.069). The differences between hairdressers within the same hair salons compared to differences between hair salons were relatively high (variance of components estimation 72 % and 28 %, respectively). When hair salons were compared based on the HI, the exposure at one hair salon (#1) differed significantly from seven out of the nine other hair salons (*P* < 0.005), with a HI of 7.5 at salon #1 compared to 0.015–2.7 at the others. The corresponding range for the exposure expressed as HI of individual hairdressers was 0.0046–13, with median of 0.90 (Fig. 2).

The chemical exposure expressed as AM HI for each hair salon was evaluated for maximum cumulative ratio (MCR). Of the ten hair salons, six were placed in substance group II (#3 and #6–10), i.e. *low concern*. The remaining four hair salons were placed in substance group I, i.e. *single substance concern*. For those hair salons, the substances of concern were formaldehyde (at hair salon #2, #4, and #5) and isopropanol (at hair salon #1). At the hair salons placed in substance group I, the exposure for individual hairdressers were placed in substance group I or II, with the exception of the exposure for one hairdresser (in hair salon #2) for whom the exposure was placed in substance group IIIB, i.e. *concern for combined effect by several substances.* For that hairdresser, the substances of concern were formaldehyde, 2,4- and 2,6- toluene diisocyanate. In two of the hair salons placed in substance group II (hair salons #9 and #10), the exposure for three hairdressers indicated elevated risk, in contrast to the average exposure for the hair salons. At hair salon #9, the exposure for two hairdressers placed in substance group I and IIIA, respectively, recalled IIIA as *concern for combined effect dominated by one substance.* At hair salon #10, the exposure for one hairdresser was also placed in substance group IIIA. For all these three hairdressers, the substance of concern was formaldehyde.

**Figure 3.**
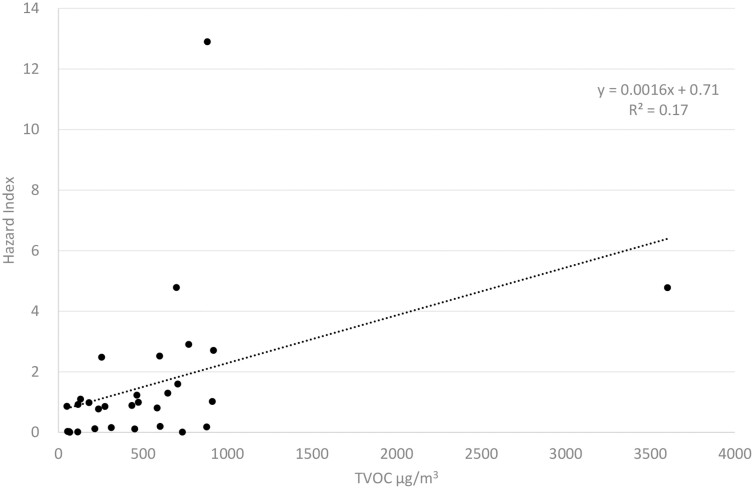
Hazard index as a function of TVOC exposure in the personal breathing zone of 30 hairdressers in Örebro, Sweden (spring 2017).

Linear regression between TVOC and HI showed positive and significant dependence (*β* = 0.002, *P* = 0.003), though the coefficient of determination was low (*R*^2^ = 0.17); see Fig. 3. The regression was also significant when the two highest values were excluded (HI=12.9 and TVOC = 3600 µg/m^3^), i.e. possible outliers.

### Indoor air climate and ventilation

For all hair salons, AM values for indoor air temperature, the concentration of CO_2_, and the relative humidity (RH) were 23.2°C, 628 ppm, and 37.9%, respectively (CV: 4.4%, 32%, and 31%, respectively). All data are presented in the supplementary material (Supplementary Table S2). The mean values for all variables were considered to be in range for normal conditions compared to expectations for Swedish dwellings which have a comfort temperature of 20–24°C, CO_2_ <1000 ppm, and seasonal RH of 30–70%. At one hair salon (#4), the mean concentration of CO_2_ was 1129 ppm, where CO_2_ >1000 ppm is considered a marker of insufficient ventilation. At three hair salons (#6, #7, and #8), conditions were slightly drier than normal, with measured RH of 23.6, 21.7, and 21.8%, respectively. Linear regression between CO_2_ and exposure showed significant and positive dependence, for both TVOC and HI (*P* = 0.028, *β* = 1.3, *R*^2^ = 0.16, and *P* = 0.016, *β* = 0.006, *R*^2^ = 0.19, respectively). Scatter plots are presented in (Supplementary Figures S3 and S4).

## Discussion

### Exposure

A considerable proportion of the hairdressers were exposed to concerning levels of airborne chemicals from hair treatment products. Calculations of the HI showed that 12 out of the 30 hairdressers had VOC exposures that equated to a HI >1. Nevertheless, HI levels at the hair salons were overall lower than previously reported in Italy (de Gennaro*et al.*, 2014), for both the share of hairdressers exposed to HI >1 and overall maximum HI. In particular, four out of 10 hairdressers were exposed to HI >1 and the maximum HI was 7.5 in the Swedish study, compared to corresponding values of 11 out of 12 and >9 in the Italian study.

The mean exposure expressed as TVOC, did not differ significantly between hair salons. In terms of HI, only one hair salon (#1) had a significantly higher HI value compared to the other salons (HI = 7.5 versus HI in the range of 0.015–2.7). Differences in exposure between individual hairdressers were suggested to have governed the results. This finding suggests that individual working procedures, defined by the combination of work tasks and individual working practices, are important determinants of exposure.

The median level of TVOC for all hairdressers, 460 µg/m^3^ (AM 520 µg/m^3^, Table 3), was higher than TVOC concentrations in Swedish small houses and apartment blocks during 1996–2005 by a factor of 2–3 times (Swedish National Board of Housing, Building, and Planning, 2010). The difference indicates a considerably higher occupational exposure for hairdressers than in private life.

Combined exposure to VOCs has also been examined in other studies of hairdressers and hair salons. In Italy, mean concentrations of TVOCs were between 279 and 3079 µg/m^3^, based on 39 VOCs measured using stationary passive sampling over 24 h during one week at 12 hair salons (de Gennaro*et al.*, 2014). The results of the present study are consistent with the lower range of TVOC concentrations reported in Italy. In 50 Portuguese hair salons, a mean concentration of TVOC of 1400 µg/m^3^ (SD 1200 µg/m^3^) measured using stationary active sampling has been reported (Mendes *et al.*, 2011), which is approximately 3-fold higher than in this study. The mean TVOC concentration in that study was based on identified and non-identified chemicals chromatographically eluting between hexane and hexadecane from stationary active sampling (airflow of 0.05 l/min) during busy working hours in 50 hair salons. In a study of 10 hair salons in Spain, TVOC concentrations were between 48 000–237 000 µg/m^3^ and 38 000–250 000 µg/m^3^ as measured by personal and stationary sampling, respectively, during working hours (Ronda *et al.*, 2009). The criteria for the definition of TVOC in the chromatogram were similar to those utilized in the Portuguese study. In a Finnish study of hair salons, TVOC concentrations were 84–465 µg/m^3^, measured by stationary passive sampling over 24 h for 2 weeks (Leino *et al.*, 1999). Those concentrations are similar to or less than the median level of TVOC in the present study. In the study from Finland, peak levels of VOC from personal exposure were also available, indicating 25 000–45 000 µg/m^3^ from a direct reading analyzer. The comparison of exposure levels between all of these studies is uncertain owing to the inclusion of different VOCs, analytical methods for chemical determination, and sampling strategies. For example, sampling during busy working hours, as in the Spanish study (Mendes *et al.*, 2011), would likely promote higher mean concentrations monitored compared to sampling during less busy working hours, as in the present study, or even higher compared to sampling over 24 h, as in the Italian or Finnish study (Leino *et al.*, 1999; de Gennaro*et al.*, 2014).

### Determinants of exposure

The measured exposure was presumed to be determined predominantly by individual working procedures combined with environmental factors. The statistically significant and positive association between measured concentrations of CO_2_ and both TVOC and HI suggested that ventilation efficiency had an effect on the exposure (Supplementary Figures S3 and S4). In addition to this, qualitative assessment of observational data provided some clues regarding their significance for measured exposure levels. At hair salon #1, the highest exposure, expressed as both HI and TVOC, was observed, but it did not coincide with any deviations of observational data concerning climate [t (°C), RH (%), and CO_2_); within range for normal conditions], performed hair treatments [in total seven haircuts, two dyeings, one bleaching] or ventilation type [natural ventilation supply air and mechanical ventilation exhaust air]. On the other hand, the individual working practices of the hairdressers remained as a probable explanation for the high exposure. Observational data at most other hair salons pointed in the same direction as for hair salon #1, i.e. no obvious impact on the exposure. Nevertheless, hair salons #4 and #8 in the end-range of measured exposure levels, similar to hair salon #1, deviated from this pattern.

At hair salon #4, where the second highest exposure was observed, it coincided with the highest CO_2_ concentration of 1129 ppm (range of the other salons 435–781 ppm), which was the only measured value above the reference concentration (1000 ppm). This finding suggests insufficient ventilation at hair salon #4, which may have had an amplifying effect on background concentrations and exposure. Stationary sampling of the background exposure could have verified or dismissed this conclusion. However, stationary sampling was not performed at any hair salon. Conversely, the lowest average exposure, observed at hair salon #8, coincided with the lowest concentration of CO_2_ at 435 ppm. This was the only hair salon featuring exhaust and supply air ventilation with heat recovery (FTX), suggesting enhanced removal of airborne pollutants. In addition, the experience among the hairdressers at hair salon #8 concerning perceived indoor air quality was consistently positive.

The impact of ventilation on chemical exposure in hair salons has been examined in previous studies. In a Norwegian study, significantly lower chemical exposure was reported in hair salons with local exhaust ventilation compared to hair salons with no ventilation (Hollund and Moen, 1998), which was consistent with a Dutch study concluding that the self-reported presence of any ventilation device was predictive of chemical exposures (Kersemaekers *et al.*, 1998). Good general ventilation has also been reported to decrease health complaints caused by hairdressing chemicals, though discomfort can be an effect of the draft (Leino *et al.*, 1999). In a study of 140 hairdressers in Shiraz, Iran, the absence of air conditioning predicted a greater reduction in lung function among the exposed (Heibati *et al.*, 2021).

### Determinant chemicals and sources

A relatively small fraction (14%) of the identified VOCs including aldehydes were considered as commonly occurring, i.e. identified in ≥10 samples (Table 3). Out of these common VOCs, a few have been reported elsewhere. Acetone, which was most frequently detected (in 96% of the samples), was also found in Taiwanese hair salons at twice the concentrations reported herein (Chang *et al.*, 2018). Limonene and nonanal were found in Italian hair salons, and were attributed to hair treatment products and traffic, respectively (de Gennaro*et al.*, 2014).

A positive correlation was observed between exposure expressed as HI and TVOC (*P* = 0.025). In other words, restricted usage of hair treatment products or other strategies to limit airborne TVOC in hair salons, may also decrease the HI and lower the risks of chemical exposure for the hairdressers. The greatest decrease in HI will occur with the reduction of chemicals with high HQs, including: formaldehyde, isopropanol, and 2,4- and 2,6-toluene diisocyanate. Among these chemicals, formaldehyde impacted the HI to the largest extent. The largest contribution to HI in Italian hair salons came from tetrachloroethylene (de Gennaro*et al.*, 2014), which was not identified in the present study.

The finding that some hairdressers have HI >1 as a result of exposure to formaldehyde or other single substances of concern shows that chemical risks can be posed at an individual level even though low risk (HI <1) is estimated for others at the same hair salon. Therefore, personal sampling is crucial for the exposure assessment of hairdressers at the individual level. Furthermore, knowledge of chemical content is fundamental so that individual working procedures can be arranged and hair treatment products can be selected to minimize exposure risks.

Identification of sources of chemicals of concern in hair salons can be troublesome. The amount of different hair treatment products, likewise their chemical content, is typically extensive and in some cases incompletely declared. In the case of formaldehyde, it can occur as a preservative ingredient or as an active ingredient in smoothing products used for straightening curly hair, so-called straighteners. For such applications, up to 0.2% formaldehyde is allowed within Europe (European Commission, 2009). In a study from Brazil, where the same content limit has been adopted, smoothing products from 23 salons were analyzed, resulting in formaldehyde concentrations between 3 and 11%, i.e. a factor of 18–54 above their national limit (Pexe *et al.*, 2019). In a case study, exposure to formaldehyde from straighteners was the reason for the development of occupational asthma in two hairdressers (Dahlgren and Talbott, 2018). Factors that influence exposure levels to formaldehyde have been found to include: the number of hairstyling and nail treatments performed (Hadei *et al.*, 2018); the number of perming treatments and workers at the salon, frequency of using formaldehyde-releasing products (Chang *et al.*, 2018); the number of customers and salon services, age of the salon, temperature (Asare-Donkor *et al.*, 2020); and concentration of formaldehyde in cosmetic products (Peteffi*et al.*, 2016). Available median or average formaldehyde concentrations in these studies were between 10 and 338 ug/m^3^ (Peteffi*et al.*, 2016; Chang *et al.*, 2018; Hadei *et al.*, 2018; Asare-Donkor *et al.*, 2020) and thereby similar to or greater than formaldehyde exposures measured in the present study (Table 3).

Isopropanol, which was a driver of the HI in one case (at hair salon #1), is a commonly occurring solvent in hair treatment products, in which it is utilized for foam inhibition, as a preservative, as a disinfectant, and for controlling viscosity. The concentration of isopropanol found in the present study (at 58 µg/m^3^) was well below the ranges of 14.5–1240 and 400–15 000 µg/m^3^ reported previously in hair salons (Hollund and Moen, 1998; Chang *et al.*, 2018).

The present study could not determine the hair treatment products that emitted 2,4- and 2,6-toluene diisocyanate, which in combination with formaldehyde also were the determinant chemicals of the HI at one hair salon (#8). Possibly, these chemicals could originate from glues used with wigs and toupées. However, to the best of our knowledge, no such work was performed during the exposure measurements. Alternatively, the exposure could have arisen from an adjacent business where eyelash extensions were done, although eyelash extensions have been reported to primarily be associated with acrylates like etyl-2-cyanoacrylate (Gunnare and Lewné, 2017).

Observed exposure profiles among the hairdressers were proposed to be further explained by examining relationships between performed hair treatments and the outcome of the MCR analysis. However, this analysis was rejected because of the limited exposure data compared to the large number of different hair treatments. Qualitatively assessed, no consequent pattern was observed between hair treatment and exposure characterized by MCR substance group I, IIIA, or IIIB. In other words, no exclusive treatment seemed to directly have caused an increased exposure risk, neither from single substances nor from combinations of substances of concern.

### Validity of the HI

In addition to the VOCs and aldehydes that were included in the present study, the hairdresser profession involves other chemicals that can potentially increase exposure risks, including hydrogen peroxide in permanent wave solution, bleaching powder and permanent dyes; thioglycolic acid and ammonia in permanent wave solution; persulfates in bleaching powder; and aromatic amines like phenylenediamine, in permanent dyes. During exposure measurements, several of the hairdressers did, in fact, perform both dyeing and bleaching as well as other treatments (Table 1). Calculation of HIs solely based on exposure to VOCs and aldehydes may lead to underestimating exposure risks. A more comprehensive sampling strategy including additional chemicals of interest would probably increase risk estimates. Nevertheless, simplified chemical sampling and analysis for screening are practical advantages compared to a more comprehensive strategy.

The chemical exposure to hairdressers occurs in mixtures of many different chemicals, particularly VOCs, but does not occur only at work. Many of the chemicals present in hair treatment products and in the air at hair salons are also present in other indoor environments, such as homes. Therefore, the exposure of hairdressers to VOCs is complex and chronic, and should be considered a cumulative exposure from different indoor environments. The HI may, for these reasons, be suitable as a risk assessment screening tool for hairdressers because it considers, by definition, chronic exposures to chemical mixtures. Application of hazard ratios based on chronic RVs instead of OELs in nonindustrial environments has been proposed elsewhere as well, e.g. in homes, schools, and offices (De Brouwere*et al.*, 2014); newly built preschools with eco-labeled building material (Persson *et al.*, 2019); beauty salons (Moradi *et al.*, 2019); and hair salons (de Gennaro*et al.*, 2014). In a study of primary schools (Mishra *et al.*, 2015), a more delimited selection of chronic RVs based only on the so-called lowest concentration of interest values (LCI values; Kephalopoulos *et al.*, 2013) was applied.

A lack of available RVs limited the accuracy of the calculated HI, as not all exposures were included in the HI. Reference and OEL were found for 18 and 21 chemicals, respectively, corresponding to 20% and 23% of the total number of identified chemicals, respectively. Approximately half of the identified chemicals were consequently excluded from the calculation of HI, which contributed to underestimates of calculated exposure risks. Identified chemicals for which OEL were utilized (instead of RVs) did also, to some extent, contribute to underestimates of risks since OEL are, in general, considerably higher than RVs. Most of the excluded chemicals (33 in total) were rarely present in the samples, but six chemicals were identified in ≥10 samples/chemical (propylene glycol, nonanal, hexadecanol, decanal, dihydromyrcenol, and hedione). Accessibility to RVs for those frequently occurring and excluded chemicals would have been of certain interest in improving the average precision of HIs for the hairdressers.

## Conclusions

A comprehensive mixture of VOCs including aldehydes was identified in the personal breathing zone of the studied hairdressers. Only a small fraction of the chemicals commonly occurred. The estimated exposure risk, expressed as an HI, was higher than one for almost half of the hairdressers. Formaldehyde made the largest single substance contribution to the estimated risk. Differences in exposure were larger between hairdressers than between hair salons. Individual working procedures, total volumetric usage, and usage of certain hair treatment products and ventilation can be important for the exposure. The HI may be suitable for screening potential exposure risks posed to hairdressers. A lack of available RVs limited the accuracy of the HI.

## Supplementary Material

wxac078_suppl_Supplementary_Material

## Data Availability

The data underlying this article are available in the article and in its online supplementary material.
