# Supplementary material for: Occupational Exposure to Volatile Organic Compounds (VOCs), Including Aldehydes for Swedish Hairdressers
Source: Ann Work Expo Health. 2022 Nov 24;67(3):366–78. doi: 10.1093/annweh/wxac078 (PMC10015803; doi:10.1093/annweh/wxac078)
Supplement: wxac078_suppl_Supplementary_Material [file wxac078_suppl_supplementary_material.pdf]

SUPPLEMENTARY MATERIAL

Occupational exposure to volatile organic compounds (VOCs), including aldehydes for Swedish hairdressers

Niklas Ricklund, Ing-Liss Bryngelsson, Jessika Hagberg

Department of Occupational and Environmental Health, Faculty of Business, Science and Engineering, Örebro University, SE 70182 Örebro, Sweden.

Correspondence to: niklas.ricklund@regionorebrolan.se

Table S1. All detected VOCs including aldehydes from measurements of exposure in the personal breathing zone of 30 hairdressers in Örebro, Sweden (spring 2017). Concentrations are given in µg/m^3 toluene equivalents. Presented RVs and OELs were extracted by the procedure described in the methods section.

Chemical	CAS No.	No. of samples detected (n=30)	Arithmetic mean	Median	Minimum	Maximum	Geometric mean	Geometric standard deviation	Standard deviation	RV(1)	OEL(2)	Data source(3)
TVOC		30	520	460	50	3600	350	2.8	640			
1-Methoxy-2-propanol	107-98-2	21	13	13	8	18	12	1.8	7.1	7000		OEHHA
1,3-Butanediol	24621-61-2	1	29	29	29	29	29					
2-Buthoxyethanol	111-76-2	3	6.7	6	4	10	6.2	1.6	3.1	1600		IRIS
2-Butoxyethylacetate	112-07-2	1	7	7	7	7	7			150		AFFSET
2-Ethylhexanol	104-76-7	9	30	3.9	2.6	220	6.9	4.4	73	540		AgBB
2-Ethylhexyl salicylate	118-60-5	2	5	5	3	7	4.6	1.8	2.8			
2-Hydroxycyclopenta-decanone	4727-18-8	2	13	12	5	20	10	2.7	11			
2-Phenoxyethanol	122-99-6	21	11	10	4	19	9.1	1.8	5.4		5700	IFA (Germany)
2-Octyl-1-dodecanol	5333-42-6	2	75	74	41	110	67	2.0	47			
2,4-Toluene diisocyanate	584-84-9	1	10	10	10	10	10				14	IFA (Sweden)
2,6-Toluene diisocyanate	91-08-7	1	12	12	12	12	12				14	IFA (Sweden)
5-Methylbenzimida-zolone	5400-75-9	1	12	12	12	12	12					
6-Methyl-5-hepten-2-on	110-93-0	2	3.5	3.5	3	4	3.5	1,.2	0.71			
9-Octadecen-1-ol	143-28-2	1	68	68	68	68	68					
Acetaldehyde	75-07-0	16	14	7.6	4.7	30	11	2.0	9.5	140		OEHHA
Acetone	67-64-1	29	53	37	11	170	42	2.0	41	70000		Health Canada
Alkane C12	13475-82-6	6	27	30	3	48	18	3.1	19		350000	IFA (Sweden)
Alkane C13-15		2	5.5	5.5	5	6	5.5	1.1	0.71		350000	IFA (Sweden)
Alkane C13-16		1	6	6	6	6	6				350000	IFA (Sweden)
Alkane C16	4390-04-9	9	31	18	3	100	16	3.4	38		350000	IFA (Sweden)
a-pinene	80-56-8	4	10	12	3	14	8.6	2.5	5	450		JRC
Benzyl acetate	140-11-4	1	26	26	26	26	26					
Benzyl alcohol	100-51-6	1	65	65	65	65	65				5000	IFA (Latvia)
Benzyl bensoate	120-51-4	1	27	27	27	27	27					
b-pinene	127-91-3	1	5	5	5	5	5				150000	IFA (Sweden)
Bisphenol A	80-05-7	1	62	62	62	62	62				2000	IFA (Sweden)
Citronellene	10281-56-8	1	3	3	3	3	3					
Cymene	527-84-4, 99-87-6, 535-77-3	2	21	21	3	39	11	6.1	25		140000	IFA (Sweden)
Decanal	112-31-2	13	9.6	8.9	5.3	22	8.7		5.1			
Decane	124-18-5	2	7.5	7.5	5	10	7.1	1.6	3.5	6000	350000	AgBB, AFFSET
Decanol	112-30-1	6	16	14	5	33	13	2.0	10		1000	IFA (Latvia)
delta-3-carene	13466-78-9, 498-15-7	3	8	9	5	10	7.7	1.5	2.6		150000	IFA (Sweden)
Diethylene glycol	111-46-6	1	5	5	5	5	5					
Diethylene glycol monododecyl ether	3055-93-4	2	10	10	6	14	9.2	1.8	5.7			
Diethyl carbitol	112-36-7	3	82	88	61	96	80	1.3	18			
Dihydromyrcenol	18479-58-8	12	8.3	7	3	20	6.8	2.0	5.4			
Dimethylphenyl carbitol-acetate	151-05-3	1	5	5	5	5	5					
Dipropylene glycol butyl ether	29911-28-2	2	7	7	7	7	7					
Dodecanol	112-53-8	22	14	7.5	3	87	8.7	2.6	19		1000	IFA (Latvia)
Etyleneglycol monododecyl ether	4536-30-5	1	10	10	10	10	10					
Eucalyptol	470-82-6	5	22	10	5	68	15	2.7	26			
Formaldehyde	59-00-0	20	13	8.8	5.3	38	11	1.9	10	9		OEHHA

Table S1 (continued).

Chemical	CAS No.	No. of samples detected (n=30)	Arithmetic mean	Median	Minimum	Maximum	Geometric mean	Geometric standard deviation	Standard deviation	RV(1)	OEL(2)	Data source(3)
Galaxolide	1222-05-5	1	4	4	4	4	4					
gamma-terpinene	99-85-4	1	25	25	25	25	25					
Geranyl acetate	105-87-3	1	4	4	4	4	4					
Geranyl acetone	3796-70-1	1	7	7	7	7	7					
Glycol ether		2	19	19	14	24	18	1.5	7.1			
Hedione	24851-98-7, 2630-39-9	10	11	5.5	3	57	6.9	2.4	16			
Heptanal	111-71-7	4	7.2	7.2	5.6	8.9	7	1.3	1.9			
Hexadecanol	36653-82-4	17	35	8	3	180	12	3.7	60			
Hexanal	66-25-1	7	7.6	6.7	5.2	10	7.3	1.3	2		40000	IFA (Poland)
Isoamyl acetate	123-92-2	3	16	16	13	20	16	1.2	3.5		500000	IFA (Sweden)
Isomethyl ionone	127-51-5	2	14	13	2	24	6	7.1	16			
Isopropanol	67-63-0	1	58	58	58	58	58			7		OEHHA
Isopropyl myristate	110-27-0	9	11	7	3	44	8.1	2.1	13			
Isopropyl palmitate	142-91-6	2	5.5	5.5	5	6	5.5	1.1	0.71			
Lauryl ethoxilate	4536-30-5	1	8	8	8	8	8					
Lilial	80-54-6	1	3	3	3	3	3					
Limonene	5989-27-5	22	45	9.5	3	310	18	4.1	70	450		JRC
Linalol	78-70-6	3	74	12	4	210	21	7.7	110			
Linalylacetat	115-95-7	4	28	10	4	85	14	3.8	39			
MEK; 2-Butanone	78-93-3	2	6	6	6	6	6			5000		IRIS
Menthol	1490-04-6	2	16	16	3	28	9.2	4.9	18			
Methyl methacrylate	80-62-6	3	9.7	10	6	13	9.2	1.5	3.5		200000	IFA (Sweden)
n-Butanol	71-36-3	1	18	18	18	18	18				45000	IFA (Sweden)
Nonane	111-84-2	1	3	3	3	3	3			200		US EPA
Nonanal	124-19-6	18	11	8.2	4.7	28	9.4	1.7	6.8			
Octinoxate	5466-77-3	1	20	20	20	20	20					
Octadecanol	112-92-5	9	75	8	5	290	23	5.2	110			
Octafluorpentyl methacrylate	355-93-1	3	30	28	4	58	19	4.0	27			
Octanal	124-13-0	5	14	12	7.4	20	13	1.5	5.8	650		AFSSET
Octyl ether	629-82-3	5	32	7	2	130	9.1	5.6	56			
Palmidrol	544-31-0, 142-78-9	2	5.5	5.5	5	6	5.5	1.1	0.71			
Propylene glycol	57-55-6	26	17	15	3	65	13	2.1	13			
Siloxanes; silicones	14857-34-2	29	200	200	5	700	100	4.3	180		2100	IFA (Denmark) (4)
Terpenes		6	22	12	4	64	13	3.0	23		150000	IFA (Sweden)
Tetradecanol	112-72-1	3	8.7	10	3	13	7.3	2.2	5.1		178000	IFA (Germany)
Tetrahydrolinalool	78-69-3	4	11	9	6	19	9.7	1.7	5.9			
Tetrahydromyrcenol	18479-57-7	3	4.7	5	4	5	4.6	1.4	0.58			
Toluene	108-88-3	6	22	22	14	31	21	1.3	6	260		WHO
Triethyl citrate	77-93-0	1	4	4	4	4	4					
Undecane	1120-21-4	2	8	8	6	10	7.8	1.4	2.8	6000		AgBB, AFSSET
Versalide	88-29-9	1	8	8	8	8	8					
Xylene	95-47-6, 108-38-3, 106-42-3	1	3	3	3	3	3			200		ATSDR

(1) Chronic reference value for health; (2) occupational 8-hour limit value; (3) acronyms: AgBB = Ausschuss zur gesundheitlichen Bewertung von Bauprodukten, Umweltbundesamt; AFSSET = L'Agence française de sécurité sanitaire de l'environnement et du travail; Health Canada = Government of Canada; IFA = Institute for Occupational Safety and Health of the German Social Accident Insurance (in parenthesis: country/work environment authority coupled to the designated OEL); IRIS = Integrated Risk Information System, US EPA; JRC = Joint Research Centre, European Commission; OEHHA = California Office of Environmental Health Hazard Assessment; (4) dimethylethoxysiloxane

Table S2. Ventilation type and results from indoor climate measurement at the 10 studied hair salons in Örebro, Sweden (spring 2017).

Salon #	Ventilation, type	t (°C)	RH (%)	CO_2 (ppm)
1	N/E	24.0	37.8	781
2	N/E	24.4	37.0	501
3	N	24.5	46.6	633
4	N/E	24.2	52.4	1129
5	N	23.2	40.8	537
6	N	21.9	23.6	629
7	N*	22.2	21.7	474
8	M	22.0	21.8	435
9	N	23.4	47.7	592
10	n/d	22.6	49.5	565
mean		23.2	37.9	628
median		23.3	39.3	578
standard deviation		1.0	11.8	201
CV		4.4%	31%	32%

N=natural ventilation; E=mechanical ventilation for exhaust air only, located at WC and/or in the staff room;

M=mechanical exhaust and supply air ventilation with heat recovery (FTX) located in the working area, WC and staff room

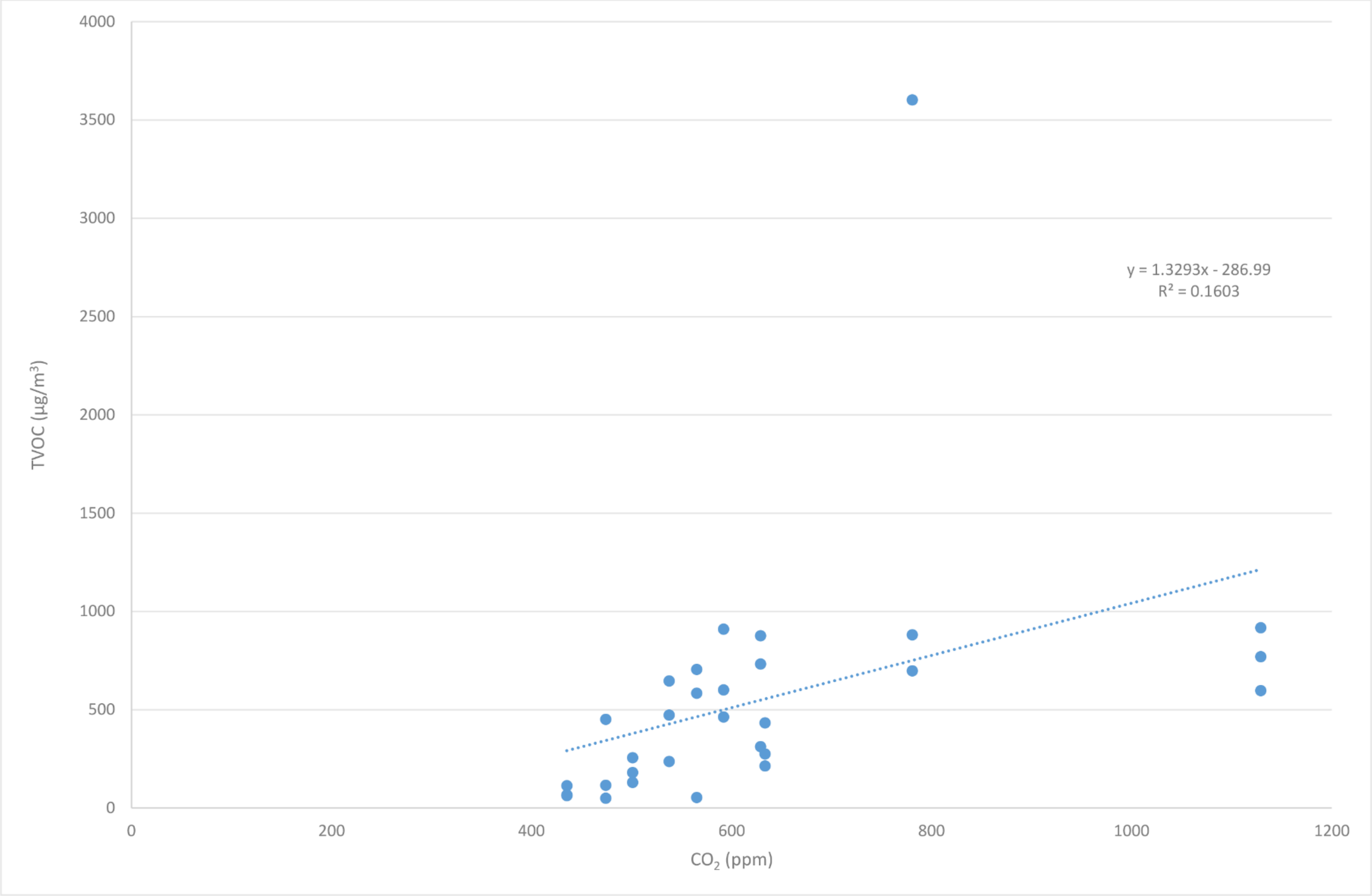

Figure S3. Exposure of hairdressers to TVOC including aldehydes as a function of CO_2 in the hair salons ($p=0.028$). Measurement were conducted in the personal breathing zone of three hairdressers at each hair salon in Örebro, Sweden (spring 2017).

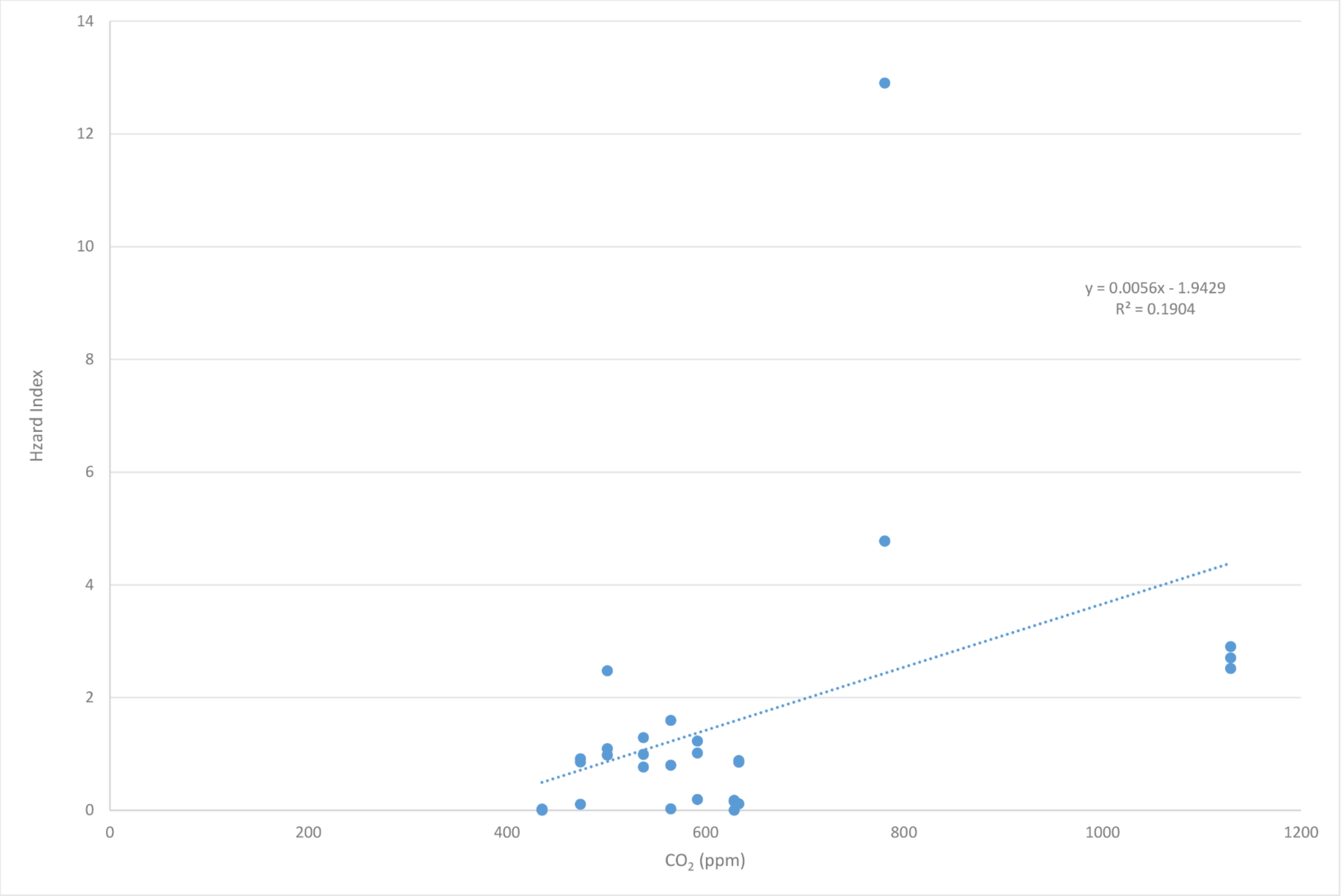

Figure S4. Hazard index of hairdressers as a function of CO_2 in the hair salons (p=0.016). Measurement were conducted in the personal breathing zone of three hairdressers at each hair salon in Örebro, Sweden (spring 2017).
